# ChatGPT has made the field of surgery full of opportunities and challenges

**DOI:** 10.1097/JS9.0000000000000454

**Published:** 2023-05-16

**Authors:** Sheng Li

**Affiliations:** Department of Anorectal Surgery, Ningbo No. 2 Hospital, Ningbo, Zhejiang, People’s Republic of China

*Dear Editor,*

Before discussing ChatGPT, it is necessary to understand its background. ChatGPT is a GPT model developed by OpenAI^1^, which is a leading language generation technology that can generate high-quality text content. OpenAI used a large amount of preprocessed and filtered text data when training the model^2,3^. In a sense, ChatGPT can be seen as an intelligent search engine.

In fact, the application of artificial intelligence (AI) in the medical field is not a novel concept, as it has existed for a long time. However, we have not paid enough attention to the potential of this field. Let us take a look at the famous Watson robot. Watson is an AI technology launched by IBM in 2011, specifically for the medical field. It can analyze a large amount of medical data and provide diagnosis and treatment suggestions for doctors based on clinical guidelines. In the clinical application of gastrointestinal tumors, Watson can provide diagnosis and treatment suggestions based on National Comprehensive Cancer Network (NCCN) and European Society for Medical Oncology (ESMO) guidelines according to the input clinical data. It is more like a quick reference manual and an AI version of the multidisciplinary team (MDT).

In the field of surgical operations, the well-known Da Vinci robotic surgical system is widely used. Some people may mistakenly believe that the surgery is entirely performed by the machine, but in fact, the surgery is still operated by the surgeon. Although robots may replace humans to perform complex surgeries in the future, the current technology cannot completely replace the clinical experience and surgical skills of doctors. With the continuous development of AI technology, its role in the field of surgery may become more significant in the future, especially in surgical planning, navigation, and support.

It is also important to note that although the diagnostic and treatment suggestions provided by ChatGPT may be valuable, they may not comply with all legal regulations. Therefore, doctors must evaluate and judge these suggestions^4^. This still requires the accumulation of clinical experience and the reading of a large amount of literature. Otherwise, it is impossible to judge the correctness of the diagnosis and treatment plan and ensure the legality of the diagnosis and treatment process^5^.

It is worth mentioning that the polishing function of ChatGPT is not just simple polishing but more like a typewriter suitable for official statements. This brings us back to the concept introduced at the beginning of this article: ChatGPT is essentially an intelligent language generation model, which is very suitable for document polishing. In fact, this article was polished multiple times using ChatGPT.

In summary, the current application of ChatGPT in the medical field has not yet had a disruptive effect, but its powerful potential is promising. This reminds me of a small story in the history of science. It is said that when Faraday demonstrated his disc generator, a noblewoman asked, ‘Mr. Faraday, what is the use of this thing?’ Faraday replied, ‘Madam, what is the use of a newborn baby?’ The current application of ChatGPT in the medical field is like a newborn baby. If it is debugged and trained through the application of a large amount of data, combined with the rapid development of AI technology, ChatGPT is very likely to become a powerful medical assistant for humans in the near future.

## Ethical approval

Not applicable.

## Consent

Not applicable.

## Sources of funding

Not applicable.

## Author contribution

L.S.: devised the concept, performed the literature search, and drafted the letter.

## Conflicts of interest disclosure

No conflict of interest.

## Research registration unique identifying number (UIN)

Not applicable.

## Guarantor

Not applicable.

## Provenance and peer review

Not applicable.

## Data availability statement

The correspondence is based exclusively on resources that are publicly available on the internet and duly cited in the ‘References’ section. No primary data were generated and reported in this manuscript. Therefore, data have not become available to any academic repository.
